# Supplementary material for: The interactome of intact mitochondria by cross-linking mass spectrometry provides evidence for coexisting respiratory supercomplexes
Source: Mol Cell Proteomics. 2017 Dec 8;17(2):216–32. doi: 10.1074/mcp.RA117.000470 (PMC5795388; doi:10.1074/mcp.RA117.000470)
Supplement: Supplemental Data [file supp_RA117.000470_133922_0_supp_23978_fzffwf.zip › spectra_annotation/mito_spectra_annotation/438-1-10-1-17-1.pdf]

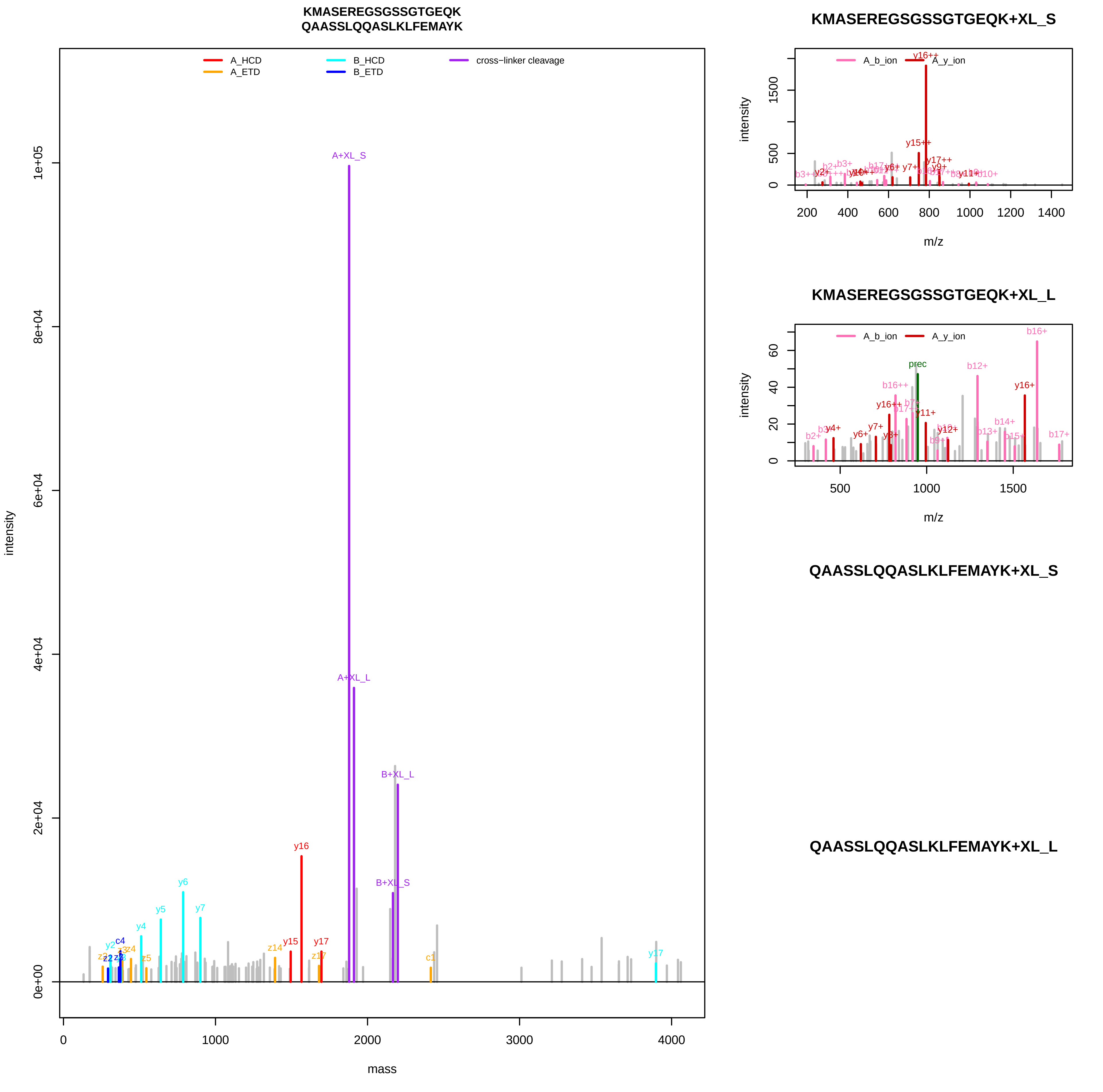
KMASEREGSGSSGTGEQK
QAASSLQQASLKLFEMAYK
A_HCD
A_ETD
B_HCD
B_ETD
cross−linker cleavage
A+XL_S
A+XL_L
B+XL_L
B+XL_S
y16
y15
y17
z14
z17
c1
y17
y4
y5
y6
y7
c4
z2
z3
z4
z5
intensity
mass
0e+00
2e+04
4e+04
6e+04
8e+04
1e+05
0
1000
2000
3000
4000
KMASEREGSGSSGTGEQK+XL_S
A_b_ion
A_y_ion
y16++
y15++
y17++
y9+
y7+
y6+
y11+
b3+
b10+
intensity
m/z
0
500
1500
200
400
600
800
1000
1200
1400
KMASEREGSGSSGTGEQK+XL_L
A_b_ion
A_y_ion
b16+
prec
b12+
y16+
b16++
y16++
y11+
y12+
b14+
b13+
b15+
b17+
b2+
y4+
y6+
y7+
intensity
m/z
0
20
40
60
500
1000
1500
QAASSLQQASLKLFEMAYK+XL_S
QAASSLQQASLKLFEMAYK+XL_L
